# The Use of Natural Agents to Counteract Telomere Shortening: Effects of a Multi-Component Extract of *Astragalus mongholicus* Bunge and Danazol

**DOI:** 10.3390/biomedicines8020031

**Published:** 2020-02-12

**Authors:** Isabelle Guinobert, Claude Blondeau, Bruno Colicchio, Noufissa Oudrhiri, Alain Dieterlen, Eric Jeandidier, Georges Deschenes, Valérie Bardot, César Cotte, Isabelle Ripoche, Patrice Carde, Lucile Berthomier, Radhia M’Kacher

**Affiliations:** 1 Groupe PiLeJe, 37 Quai de Grenelle, 75015 Paris Cedex 15, Naturopôle, Les Tiolans, 03800 Saint-Bonnet de Rochefort, France; i.guinobert@pileje.com (I.G.); c.blondeau@pileje.com (C.B.); v.bardot@pileje.com (V.B.); c.cotte@pileje-industrie.com (C.C.); 2 IRIMAS, Institut de Recherche en Informatique, Mathématiques, Automatique et Signal, Université de Haute-Alsace, 68093 Mulhouse, France; bruno.colicchio@uha.fr (B.C.); alain.dieterlen@uha.fr (A.D.); 3 Service d’Hématologie Moléculaire et Cytogénétique Paul Brousse CHU Paris Sud, Université Paris Sud, Inserm UMRS935, 94800 Villejuif, France; noufissa.oudrhiri@aphp.fr; 4 Service de Génétique Médicale, Groupe Hospitalier de la Région de Mulhouse et Sud-Alsace, 68070 Mulhouse, France; jeandidiere@ghrmsa.fr; 5 Service de Néphrologie, APHP-Hôpital Robert Debré, 75019 Paris, France; georges.deschenes@aphp.fr; 6 Institut de Chimie de Clermont-Ferrand, Université Clermont Auvergne, CNRS, SIGMA Clermont, BP 10448, 63000 Clermont-Ferrand, France; isabelle.ripoche@sigma-clermont.fr (I.R.); lucile.berthomier@sigma-clermont.fr (L.B.); 7 Département d’hématologie, Gustave Roussy Cancer Campus, université Paris Saclay, 94808 Villejuif, France; dr.pcarde@gmail.com; 8 Cell Environment, DNA damage R&D, 75020 Paris, France

**Keywords:** *Astragalus mongholicus* Bunge, danazol, telomere, telomerase, aging

## Abstract

A link between telomere shortening and oxidative stress was found in aging people and patients with cancer or inflammatory diseases. Extracts of *Astragalus* spp. are known to stimulate telomerase activity, thereby compensating telomere shortening. We characterized a multi-component hydroethanolic root extract (HRE) of *Astragalus mongholicus* Bunge and assessed its effects on telomeres compared to those of danazol. Astragalosides I to IV, flavonoids, amino acids and sugars were detected in the HRE. Samples of peripheral blood lymphocytes with short telomeres from 18 healthy donors (mean age 63.5 years; range 32–86 years) were exposed to a single dose of 1 µg/mL HRE or danazol for three days. Telomere length and telomerase expression were then measured. Significant elongation of telomeres associated to a less toxicity was observed in lymphocytes from 13/18 donors following HRE treatment (0.54 kb (0.15–2.06 kb)) and in those from 9/18 donors after danazol treatment (0.95 kb (0.06–2.06 kb)). The rate of cells with short telomeres (<3 kb) decreased in lymphocytes from all donors after exposure to either HRE or danazol, telomere elongation being telomerase-dependent. These findings suggest that the HRE could be used for the management of age-related diseases.

## 1. Introduction

The age structure of human populations is changing, with an increase in the proportion of elderly individuals [1]. Age-related diseases are currently the most important causes of morbidity and mortality worldwide [2,3]. There is consequently an urgent need to develop approaches conducive to better health in the elderly [4], thereby improving their quality of life and reducing medical costs [5,6].

Telomeres are dynamic nucleoprotein structures that protect the ends of chromosomes from degradation and activation of the DNA damage response. Telomeres are considered to be a biological clock, playing a major role in aging and genome stability [7]. It is now well-documented that telomere dysfunction is a potential biomarker for age-related diseases and can contribute to the prognosis of several diseases [8,9,10,11,12]. Telomere sensitivity to inflammation and oxidative stress such as ionizing radiation has been previously demonstrated [13]. This sensitivity promotes telomere shortening and replicative senescence [14] leading to chromosomal instability [15]. This phenomenon was found to occur in both proliferative and nonproliferative tissues [16]. To overcome telomere dysfunction, activation of a telomere maintenance mechanism is required to support cell proliferation and immortalization. In most cases, telomeres are elongated by telomerase, a cellular reverse transcriptase capable of compensating telomere shortening through de novo addition of (T2AG3)n [17]. However, telomerase activity can decline with age [18]. Telomerase reactivation approaches have been investigated to counteract telomere shortening and its consequences and have consequently been proposed for the treatment of age-related diseases and telomeropathies [19]. It was shown that antioxidant and anti-inflammatory agents can be used to slow the loss of telomere length [20]. Furthermore, several studies have reported telomere elongation by androgens such as danazol [7,21] and by herbal products [22,23,24,25,26,27]. 

In traditional Chinese medicine, *Astragali Radix* (known as Huang Qi), the dried root of *Astragalus membranaceus* (Fisch.) Bunge or *Astragalus mongholicus* Bunge, is an herbal medicine that has been used to counteract oxidative stress, inflammation and aging since ancient times [28,29]. Pharmacological studies have shown that *Astragalus* spp. extracts and their principal components (saponins, flavonoids and polysaccharides) act on aging via several mechanisms [23,30]. In addition to their antioxidant, anti-inflammatory, immunoregulatory and anticancer effects, extracts of *Astragalus* spp. have been shown to exert beneficial effects on telomeres and to stimulate telomerase activity in various models [22,23,31]. Most studies investigated cycloastragenol (TA-65), a single chemical entity isolated by a proprietary purification process from a root extract of *Astragalus membranaceus* [22,24,25,31,32].

The objective of this study was to determine the phytochemical composition of a multicomponent hydroethanolic root extract (HRE) of *Astragalus mongholicus* Bunge and to assess its effects on telomere length and telomerase expression in lymphocytes from healthy donors with short telomeres, per comparison with those of danazol, no such comparison having been made up to now within the same cohort. We demonstrated significant telomere elongation in lymphocytes exposed to HRE, associated with less toxicity than that induced by danazol treatment. This telomere elongation could be related to telomerase activation. The proportion of lymphocytes with short telomeres decreased significantly after exposure to either HRE or danazol in samples from all donors. 

## 2. Materials and Methods 

### 2.1. Preparation of the Hydroethanolic Root Extract of Astragalus mongholicus Bunge

Roots of *A. mongholicus* Bunge were collected in China in October 2015 and identified by Gilles Thébaud from the UniVegE service of the University of Clermont-Ferrand (France) in which a voucher specimen was deposited (CLF110821). CLF is registered in the Index Herbariorum of the New York Botanical Garden.

The liquid HRE of *A. mongholicus* Bunge evaluated in this study was produced by PiLeJe Industrie (France) according to the patented process WO2001056584A1. The batch used in this study (no. C-16K404) contained 30.7% of dry material containing 0.05% formononetin and 0.16% of astragaloside IV. The drug extract ratio (DER) of HRE, expressed as the ratio of the dry weight of the original fresh plant material to that of the resulting extract, was 3:1. After addition of glycerol, the *A*. *mongholicus* Bunge HRE corresponds to a formononetin-standardized extract of *A. mongholicus* Bunge (EPS Astragale, PiLeJe Laboratoire, France).

### 2.2. High-Performance Thin-Layer Chromatography (HPTLC) Analysis of A. mongholicus Bunge HRE

Standards were diluted in methanol at a concentration of 0.1 mg/mL for formononetin (Extrasynthèse, Genay, France) and 0.51 mg/mL for astragaloside IV (European Directorate for the Quality of Medicines & HealthCare, Strasbourg, France). *A. mongholicus* Bunge HRE without glycerol (4 mL) was diluted in 16 mL of a mixture of ethanol and water (50/50:*v*/*v*). The resultant solution was shaken and centrifuged for 3 min at 4400 rpm. The supernatant solution was transferred into individual vials and then subjected to HPTLC analysis.

HPTLC analysis was performed on 100.0 × 100.0 mm silica gel 60 F 254 HPTLC glass plates (Merck, Germany). Standard solutions and samples were applied to the plates in bands 8.0 mm wide using a CAMAG Automatic TLC sampler (ATS 4). The plates were developed in a CAMAG Automatic developing chamber (ADC2), derivatization being accomplished using a TLC plate heater and a CAMAG Chromatogram Immersion Device. The chromatograms were recorded by a CAMAG Visualizer with WinCATS software. The specific chromatographic conditions used for the three types of compounds analyzed are presented in Supplementary Table S1.

### 2.3. Liquid Chromatography/Mass Spectrometry (LC/MS) Analysis of A. mongholicus Bunge HRE

Ultra-high performance liquid chromatography (UHPLC) analyses were performed on an Ultimate 3000 RSLC UHPLC system (Thermo Fisher Scientific Inc., Waltham, MA, USA) coupled with a binary pump (U3000 HPG-3400RS) and a diode array detector. Compounds were separated on an Uptisphere Strategy C18 column (25 × 4.6 mm, 5 μm; Interchim, Montluçon, France) and maintained at 40 °C. The flow rate was 0.8 mL/min, and the injection volume was 5 µL. Mobile phases were phase A, 0.1% (*v*/*v*) formic acid in water and phase B, 0.1% (*v*/*v*) formic acid in acetonitrile with the linear gradient: 0–25 min, 100%–0% of phase A. The UHPLC system was connected to a Q-Exactive Orbitrap (Thermo Fisher Scientific Inc.) mass spectrometer operated in negative and positive electrospray ionization modes. Source operating conditions were: 3 kV spray voltage for the negative mode and 3.5 kV spray voltage for the positive mode; 320 °C heated capillary temperature; 475 °C auxiliary gas temperature; sheath, sweep and auxiliary gas (nitrogen) flow rate 60, 18 and 4 arbitrary units, respectively, and collision cell voltage between 20 and 50 eV. Full-scan data were obtained at a resolution of 35,000, whereas tandem mass spectrometry (MS^2^) data were obtained at a resolution of 17,500. Data were processed using Xcalibur software (Thermo Fisher Scientific Inc.). 

The components of the HRE were characterized according to their retention times, mass spectral data and comparison with authentic standards, when available, or otherwise with published data.

### 2.4. In Vitro Exposure of Cell Lines And Cytotoxicity Approach

Lymphoblastoid cells from two human cell lines (BJAB and DG-75) were treated with increasing doses of HRE (0.01, 0.1, 1 and 10 µg/mL) dissolved in ethanol 30% for 72 h at 37°C. For the controls, the same procedure was followed with ethanol alone (negative control) and with danazol (Sigma, Saint Quentin Fallavier, France; dissolved in DMSO to concentrations of 0.01, 0.1, 1 and 10 µg/mL; positive control). Survival was assessed using trypan blue, cell proliferation being evaluated on the basis of the mitotic index after cell arrest. The number of cells in metaphase and interphase were scored. The mitotic index was the ratio between the number of cells in metaphase to the total number of scored cells.

### 2.5. Peripheral Blood Lymphocyte In Vitro Exposure and Culture Conditions

Peripheral blood lymphocytes from 18 healthy donors (15 men and 3 women) with a mean age of 63.5 years (range 32–86 years) were used in this study. A large cohort of 150 healthy donors was used as a control. The use of samples from healthy donors has been approved by the Ethic Committee of Gustave Roussy Cancer Campus University Paris Saclay (ethical approval code: Comités de protection des personnes CPP 97/06, and Ile-de-France/ Nephrovir/ 2010). Informed consent was obtained from all donors included in this study. 

Blood lymphocytes were exposed to 1 µg/mL HRE or danazol and cultured in RPMI 1640 medium (Gibco-BRL, Grand Island, NY, USA) supplemented with Glutamax, 10% fetal bovine serum (Eurobio, Courtaboeuf, France) and antibiotics (penicillin and streptomycin; Gibco-BRL) for 72 h (3 days) at 37 °C. The effects of *A. mongholicus* Bunge HRE and danazol on lymphocyte proliferation and telomere length were assessed. 

### 2.6. Telomere Quantification

Telomeres were quantified in interphase cells using the quantitative fluorescence in situ hybridization (Q-FISH) technique with a Cy-3-labelled PNA probe specific for TTAGGG (Eurogenetec, Liège, Belgium), permitting investigation of intercellular variation in a large number of scored cells. The detailed procedure was described previously [11,12]. Quantitative image acquisition and analysis were performed using Metacyte software (Metasystem, version 3.9.1; Altlussheim, Germany). The mean fluorescence intensity (FI) of telomeres was automatically quantified in 10,000 nuclei on each slide. Settings for exposure and gain remained constant between captures. The experiments were performed in triplicate. Internal (cell line) and external (fluorescence beads) controls of the fluorescence intensity were used in each experiment. Telomere length, measured as mean FI, was also translated into the mean telomere length in kilobases (kb) using a standard curve performed in cancer patients, as well as in human cell lines using the telomeric restriction fragment (TRF) [11] (Supplementary Figure S1). Mean telomere length was expressed in kb.

### 2.7. Telomerase Expression Using Immunofluorescence 

To quantify telomerase expression, peripheral blood lymphocytes of 6 donors were isolated in Ficoll medium (Ficoll, Biochrom AG, Berlin, Germany) and then cultured in RPMI medium (Gibco-BRL) supplemented with 10% fetal bovine serum (Eurobio, Courtaboeuf, France) and antibiotics (penicillin and streptomycin; Gibco-BRL) at 37 °C for 72 h. The detailed procedure was published previously [33]. 

### 2.8. Statistical Analysis

All data were analyzed using R software version 3.5.3 and libraries. Mean comparisons were computed using the two-sample Wilcoxon test. The following convention for symbols indicating statistical significance were used: ns for *p* > 0.05, * for *p* ≤ 0.05, ** for *p* ≤ 0.01, *** for *p* ≤ 0.001 and **** for *p* ≤ 0.0001. The regression curve presented was computed on the mean telomere length previously determined in a cohort of 150 healthy donors [34] using a linear regression model (lm).

## 3. Results

### 3.1. Characteristics of A. mongholicus Bunge HRE

**Figure 1:**
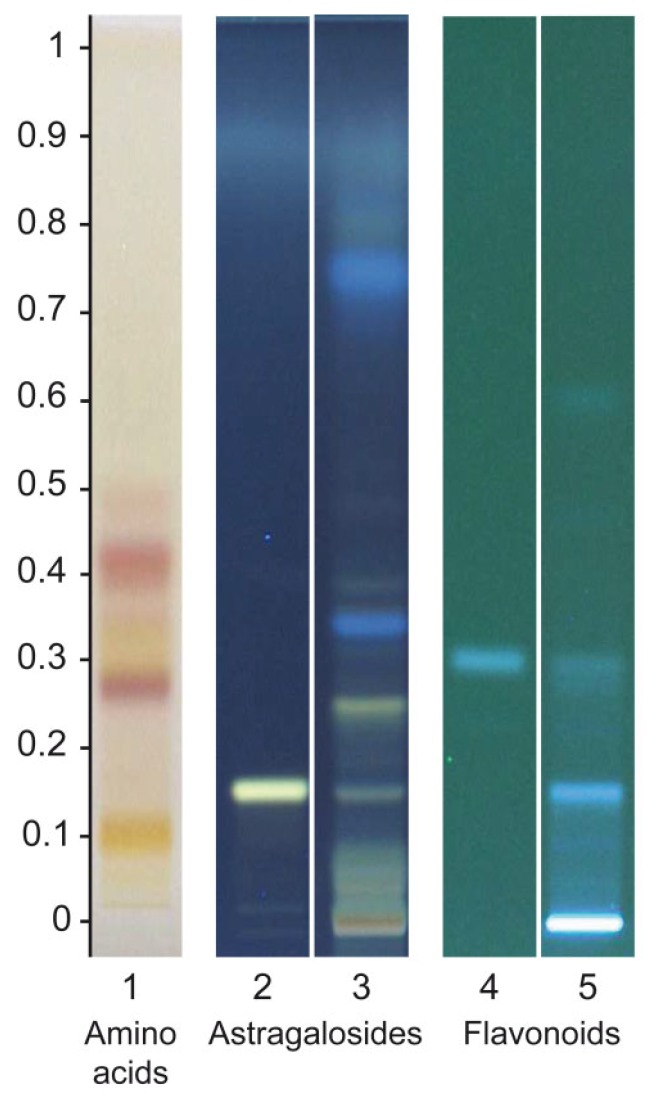
High-performance thin-layer chromatography (HPTLC) plate for amino acids, astragalosides and flavonoids. Track 1: *A. mongholicus* Bunge HRE (0.2 µL), Track 2: astragaloside IV (2 µL), Track 3: *A. mongholicus* Bunge HRE (1 µL), Track 4: formononetin (4 µL) and Track 5: *A. mongholicus* Bunge HRE (3.4 µL).

HPTLC analysis revealed the presence of astragalosides, including astragaloside IV, flavonoids, including formononetin, and amino acids in the *A. mongholicus* Bunge HRE (Figure 1). Analysis by UHPLC-MS confirmed the presence of astragaloside IV and formononetin, additionally revealing the presence of astragalosides I, II and III (Supplementary Figure S2 and Tables S2 and S3). Isoflavones, including calycosin, ononin and calycosin-7-o-β-d-glucoside, were also detected. Various amino acids were identified, including L-canavanine, asparagine, aspartic acid, glutamic acid, leucine and phenylalanine. 

### 3.2. HRE Cytotoxicity

The cytotoxicity of HRE and danazol were assessed on the basis of cell viability and mitotic index on two human cell lines (BJAB and DG-75) and human peripheral blood lymphocytes. 

**Figure 2:**
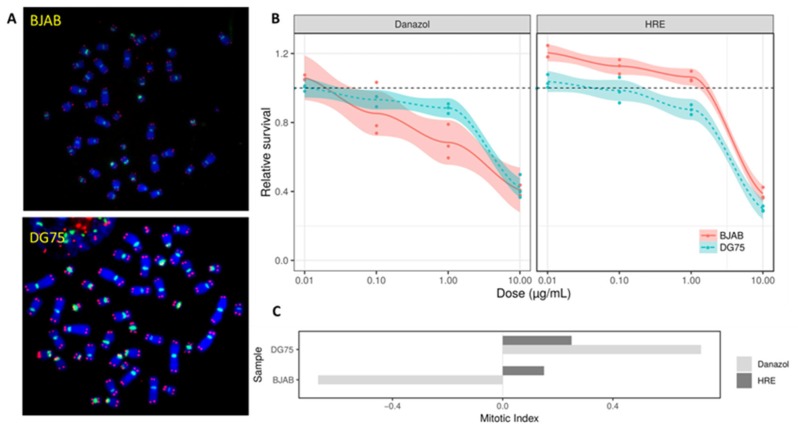
Toxicity of hydroethanolic root extract (HRE) and danazol. BJAB and DG-75 cells were incubated with increasing doses of the HRE or danazol for 3 days. (**A**) Metaphases from BJAB and DG75 cell lines stained with a Cy3 telomere probe (red) and FITC centromere probe (green) showing drastically short telomeres in BJAB cells. (**B**) Cell viability determined by manual cell counting after staining with trypan blue. Relative survival was calculated after in vitro exposure to the HRE or danazol. The data are representative of three independent experiments and expressed as the mean ratio ± standard error of the mean. The experiments were performed in triplicate. (**C**) Mitotic index variation after exposure to HRE and danazol for 3 days following a single dose of 1 µg/mL. Two slides were used for each condition.

The choice of these cell lines was based on their telomere status, the BJAB cell line being characterized by drastically short telomeres compared to the DG-75 cell line (Figure 2A). Cell viability following exposure to each of the four HRE and danazol doses was assessed by manual cell counting using trypan blue to stain dead cells (Figure 2B). No significant difference in cell viability was observed between cells treated with HRE at 1 µg/mL and those treated with the vehicle (control). Interestingly, BJAB cell viability was significantly higher after treatment with HRE at 0.01 µg/mL than in the controls (*p* < 0.01). In contrast, danazol 1 μg/mL induced more toxicity in BJAB cells than in DG75 cells, showing that BJAB cells are more sensitive to danazol than DG75 cells.

Similarly, the mitotic index was evaluated after the HRE and danazol treatments and compared to the control value in the cell lines (Figure 2C). With regard to the BJAB cell line, the mitotic index after HRE treatment was higher than that in the controls (more than 10.3%), confirming the high cell viability results obtained after HRE treatment. In contrast, the mitotic index was reduced in BJAB cells treated with danazol and confirmed the higher sensitivity of these cells to danazol. The results of these experiments permitted selection of an optimal dose for human lymphocyte exposure that did not induce increased toxicity, namely 1 µg/mL for both the HRE and danazol.

**Figure 3:**
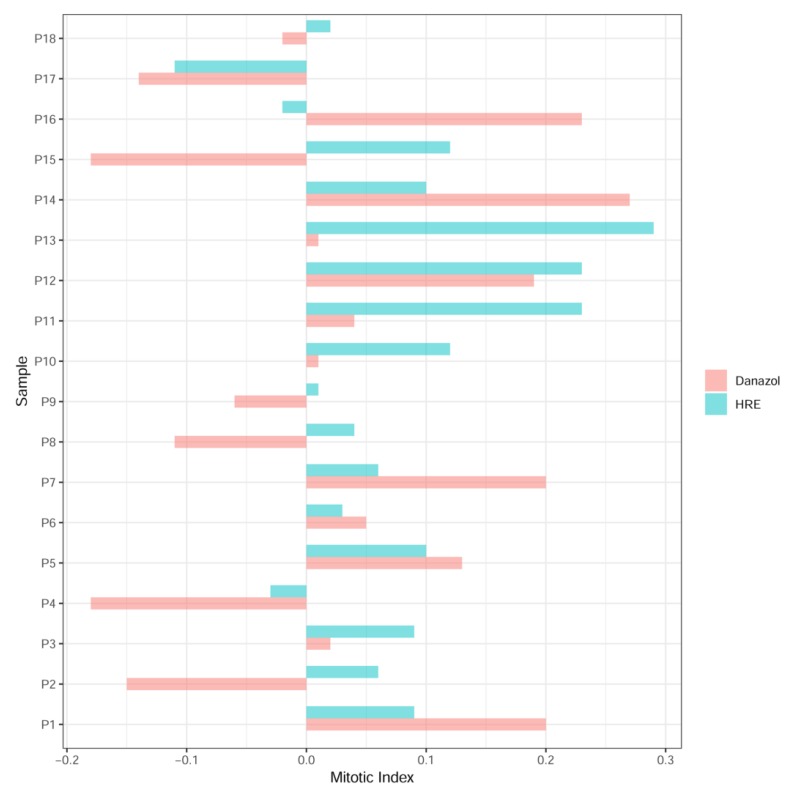
Toxicity of HRE and danazol treatment on circulating lymphocytes from healthy donors based on the relative change in the mitotic index after HRE and danazol treatment (1 µg/mL) compared to control values. Two slides were used to establish the mitotic index for each condition.

Mitotic index was also evaluated in peripheral blood lymphocytes from 18 healthy donors before and after exposure to 1 µg/mL of HRE or danazol during three days. Figure 3 shows the relative change in the mitotic index after HRE and danazol treatments compared to the control. A higher mitotic index was observed in 15 donors after HRE treatment versus 11 donors after danazol treatment.

### 3.3. In Vitro Exposure of Peripheral Blood Lymphocytes to HRE Induced Telomere Elongation and Decreased the Proportion of Cells with Short Telomeres 

**Figure 4:**
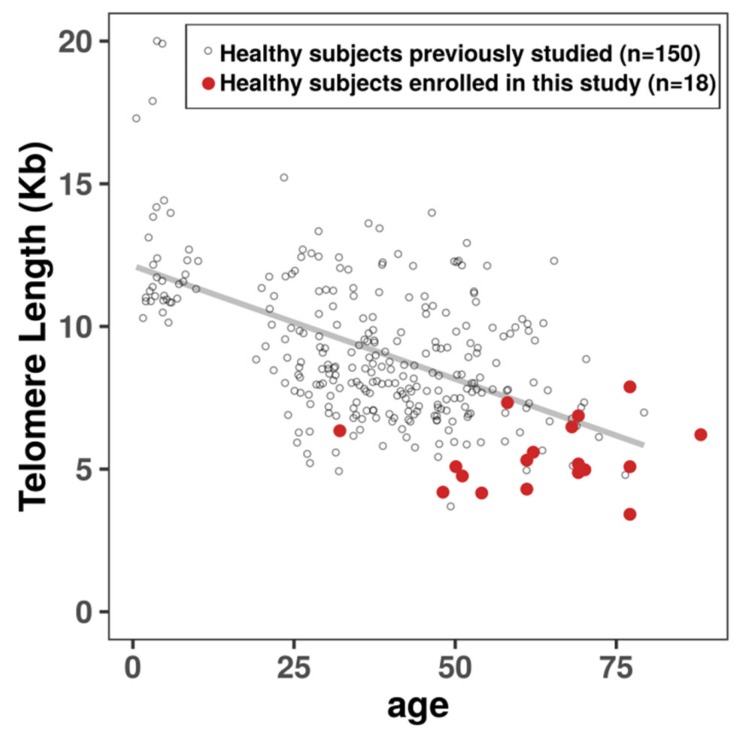
Telomere length in peripheral blood lymphocytes as a function of age. The healthy blood donors enrolled in this study presented very short telomeres in their peripheral blood lymphocytes compared to those of a previously studied cohort of 150 blood donors [34]. The regression line indicates natural telomere shortening with age (79 pb per year; Y = 12.1–0.79 and R2 = 0.29). Telomere length was measured by Q-FISH. Fluorescence intensity was transformed to kilobases according to the correlation between Q-FISH and Southern Blot results.

Telomere length was measured using the quantitative fluorescence in situ hybridization (Q-FISH) technique in interphase cells, permitting the investigation of intercellular variation in a large number of scored cells. The mean telomere length in peripheral lymphocytes from each donor was based on the quantification of telomere signal intensity performed in triplicate. We first analyzed telomere length in a large cohort of healthy donors spanning a large age range (150 healthy donors; 2–76 years). The rate of telomere loss was calculated from the decrease in telomere length as a function of donor age. Telomere length declined at a rate of 79 pb/year (*p* < 10^−6^, R2 = 0.29; Figure 4). 

To assess the effect of HRE and danazol on telomere length, 18 healthy donors (mean age 63.5 years; range 32–86 years) with short telomeres were selected. These donors had a mean telomere length of 5.97 kb (3.47–7.94 kb; Figure 4). Telomere length in peripheral blood lymphocytes from 15 of these donors was lower than the median age-dependent telomere length (Figure 4).

**Figure 5:**
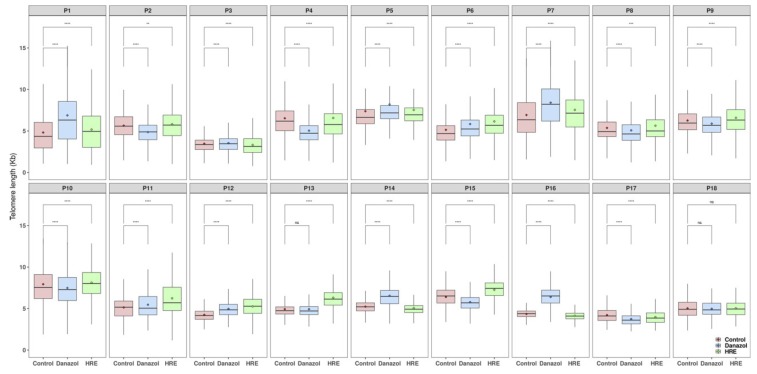
Box plots of telomere length in circulating lymphocytes from healthy donors determined by Q-FISH. Mean values are shown by diamond-shaped points. The middle line reflects the median, the box length reflects the interquartile range (interquartile range, 75th–25th percentiles) and the whiskers reflect the 5th and 95th percentiles. Statistical significance of the difference between telomere length of circulating lymphocytes before and after treatment with HRE and danazol (1 µg/mL) (one-way analysis of means ANOVA): **** *p* < 0.0001. NS: not significant.

After exposure to HRE, significant telomere elongation was observed in blood lymphocytes from 13 of the 18 donors (72%, 0.54 kb (0.15–2.06 kb); p < 0.001; Figure 5).

**Figure 6:**
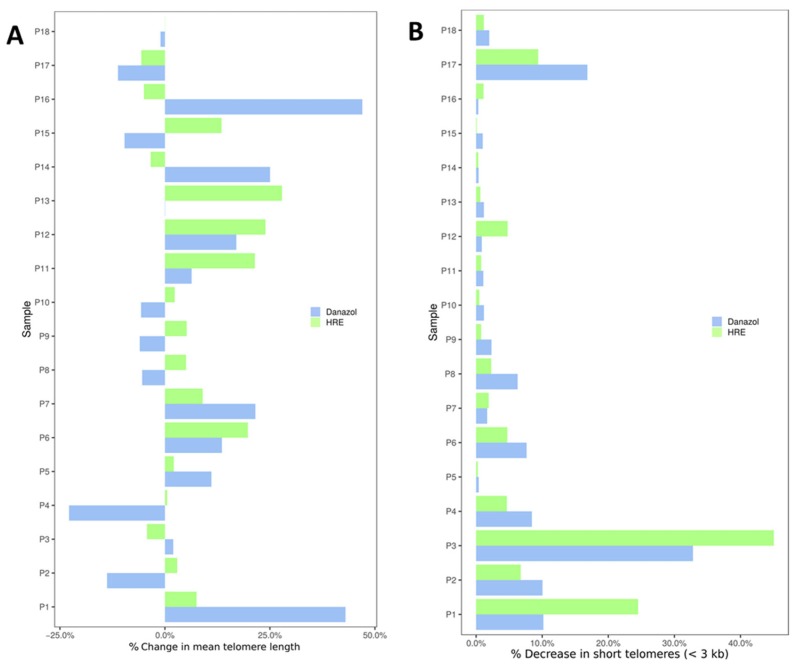
Changes in telomere length in circulating lymphocytes from each healthy donor after exposure to HRE and to danazol (1 µg/mL). (**A**) Change in mean telomere length and (**B**) decrease in the proportion of lymphocytes showing drastic telomere shortening (<3 kb).

The increase in telomere length compared to that measured before treatment varied from 5% to 27% (Figure 6A). No significant change in telomere length was observed in lymphocytes from one donor, and moderate toxicity following exposure to HRE was seen in lymphocytes from four donors (Figure 5 and Figure 6A). We have also analyzed the rate of cells with very short telomeres, less than 3kb [35]. The choice of this telomere length was based on both the telomere length observed in patients with genetic disorders related to telomere mutations, such as dyskeratosis congenita and aplastic anemia [36], and the telomere length in the cohort used in our study (mean telomere length: 5.97 kb). After HRE exposure, a decrease in the proportion of cells with very short telomeres (less than 3 kb) was observed in lymphocytes from all donors (Figure 6B and Supplementary Figure S3). 

After danazol exposure, significant telomere elongation was observed in cultured circulating lymphocytes from 9 of the 18 donors (50%, 0.95 kb (0.06–2.06 kb); p < 0.001; Figure 5). The increase in telomere length varied from 2% to 42% (Figure 6A). No significant change was observed in lymphocytes from two donors, and toxicity following exposure to danazol was seen in lymphocytes from seven donors (Figure 5 and Figure 6A). As seen with HRE, a decrease in the rate of cells with very short telomeres (less than 3 kb) was observed in lymphocytes from all donors (Figure 6B and Supplementary Figure S3). Comparison of the effects of HRE and danazol on telomere elongation showed less toxicity and a positive response in lymphocytes from more donors following exposure to HRE (13/18) than after exposure to danazol (9/18). In a large cohort of 150 healthy donors, we determined the natural shortening of telomeres related to natural aging to be 79 pb/year [33]. The mean telomere elongation corresponded to a gain of 6.77 years of age (0.54 kb) after HRE and 12.08 years (0.95 kb) after danazol treatment.

### 3.4. The HRE Induced Telomerase Expression and Led to Telomerase-Dependent Elongation 

**Figure 7:**
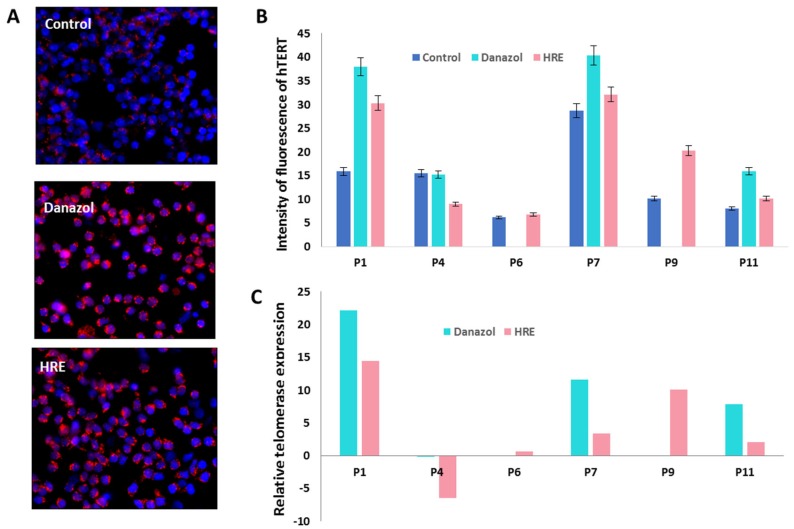
Telomerase expression after exposure to HRE and to danazol. (**A**) Representative image of telomerase reverse transcriptase (hTERT) expression (red) after in vitro treatment of circulating lymphocytes from healthy donors using immunofluorescence staining. Cells were counterstained with DAPI (blue) (**B**) Quantification of the intensity of fluorescence of hTERT protein; 10,000 cells were scored. All data are representative of three independent experiments and are expressed as the mean ± standard error of the mean. The experiments were performed in triplicate. (**C**) Histogram displaying the change in telomerase expression in circulating lymphocytes from healthy donors after exposure to HRE and danazol as a multiple of telomerase expression before exposure to these agents. The absence of hTERT expression for P6 and P9 after danazol treatment was due to a technical issue.

Previously, we demonstrated that telomerase reverse transcriptase (hTERT) expression, assessed by immunofluorescence, was strongly correlated with telomerase activity measured by the telomeric repeat amplification protocol (TRAP) assay [33]. In this study, hTERT expression was assessed using immunofluorescence staining in six donors. A significant increase in hTERT expression was observed after exposure to HRE (0.6- to 14.4-fold relative to control) or to danazol (7.8- to 22.2-fold relative to control; Figure 7). The increase in hTERT expression observed after exposure to HRE was lower than that observed after exposure to danazol.

## 4. Discussion

Aging is a complex and multifactorial process. Telomere shortening and dysfunction, with other factors such as oxidative stress and replicative senescence, play a major role in natural aging and age-related diseases [1]. The implication of telomere dysfunction related to genetic susceptibility and/or environmental factors in genomic instability and age-related diseases has been clearly demonstrated [11,12,37]. The development of novel specific and effective agents devoid of major systemic side effects is a new challenge in the battle against aging. Telomere shortening, the relevant marker of aging, is also responsible for cellular and organismal aging. Counteracting telomere attrition and its consequences has been a main target for research during the last decade [7,22]. Regenerative medicine has also focused on strategies to maintain telomere length [31]. Androgens, such as danazol, have been used to promote telomere elongation in patients with telomeropathies or telomere syndromes [21]. Danazol has also been used to treat bone marrow syndromes for decades with mixed responses and without knowledge of the underlying mechanisms. Recently, several studies have demonstrated that danazol regulates expression of the telomerase gene in both in vitro and in animal models [38,39]. However, the results concerning telomere elongation after in vivo treatment with danazol have been inconsistent, with substantial variation between patients and evidence of poor tolerance [40]. In addition to androgens, several herbal products have been tested for their effects on telomeres [23,25,26,28]. Cycloastragenol (TA-65), a bioactive compound isolated from *Astragalus membranaceus*, has been shown to increase telomere length and activate telomerase in both preclinical and clinical studies [22,24,25,31,32,41,42]. 

In our laboratory, telomere length has already been evaluated in large cohorts of healthy donors and cancer patients [11,33,34,35]. In a large cohort of healthy donors, telomere length decreased at a rate of 79 pb per year, which is in line with the rate published by other authors [43,44,45]. In this study, we assessed the effects of a multi-component extract of *Astragalus mongholicus* Bunge on telomere elongation and telomerase activity in comparison to those of danazol. Up to now, no such comparison had been performed within the same cohort.

The first step in this study consisted to assess the toxicity of HRE using human cell lines. Less toxicity was observed with HRE than with danazol in terms of cellular viability and proliferation (mitotic index). These data confirmed the toxicity of danazol, which had been described previously, mainly apparent in the context of drastically short telomeres [21]. The BJAB cell line, characterized by very short telomeres, showed higher proliferation after exposure to low doses of HRE. In contrast, there was a significant decrease in cellular proliferation and the mitotic index in BJAB cells after danazol treatment. This discrepancy in the response of BJAB cells to danazol and HRE treatment could be due to the telomere shortening in these cells [46]. Additional investigations are needed to elucidate the mechanisms underlying the sensitivity of BJAB cells to treatment.

The effects of HRE and danazol on telomere elongation and telomerase expression in cultured lymphocytes were evaluated using a specific cohort of healthy blood donors presenting short telomeres essentially related to their age. We clearly demonstrated that exposure to HRE induced significant telomere elongation in lymphocytes from more than 70% of the donors and reduced the proportion of lymphocytes with short telomeres (below 3 kb) from all donors, with very moderate toxicity. This telomere elongation was related to an increase in telomerase activity. These data are in line with those of previously published studies on cycloastragenol [32,41,42]. 

We also showed that exposure to HRE not only achieved a telomere elongation corresponding to 6.8 years of natural aging but also decreased the proportion of cells with very short telomeres (less than 3 kb) from all the donors. These cells with drastically short telomeres are those that could be at the origin of a number of malignancies and age-related diseases [8]. The results obtained with danazol are in line with those obtained in patients with bone marrow syndromes [21], as well as in those with telomeropathies [40]. In addition, a broader heterogeneity in the responses and a higher rate of toxicity in lymphocytes of 39% (7/18) of the donors has been observed with danazol as compared to that seen with HRE. Similarly a broad heterogeneity of clinical responses has been observed in aplastic anemia in terms of response to danazol [47]. 

The extract tested in this work was obtained from roots of *Astragalus mongholicus* Bunge according to a patented process preserving the integrity and diversity of the plant totum, defined as the entire set of compounds contained in the part of the plant used for extraction. Phytochemical analysis of the *A. mongholicus* Bunge HRE tested revealed the presence of formononetin and astragaloside IV. Isoflavones, including calycosin, ononin and calycosin-7-o-β-d-glucoside, were also detected. In a study performed with a multi-component extract of *Astragalus membranaceus* roots [31], mild increases in telomerase were observed in a few donor T cell cultures compared to cycloastragenol, which significantly increased telomerase activity. In this study, the extract tested was “an extract of the root of *Astragalus membranaceus* but not a single purified compound entity”. No other information on the composition of the extract is provided. In another study, an *Astragalus* extract (also without further specifications) was reported to induce significant telomerase activity in primary human IMR90 cells [25]. Astragaloside IV identified in our extract is most certainly responsible for at least some of the effects observed in our study. To our knowledge, no studies have been carried out with astragaloside IV alone. Cycloastragenol, with which most studies have been conducted and the effects observed, is a derivative of astragaloside IV. No data exist on the other compounds identified in our extract regarding a possible effect on telomeres and telomerase. However, it cannot be excluded that some of them are active. This remains to be investigated. In addition, compounds that were not detected in the HRE in the analyses performed, but that could still be present, could also be involved. For example, two isomers of 4-hydroxy-5-hydroxymethyl-(1,3)dioxolan-2,60-spirane-50,60,70,80-tetrahydro-indolizine-30-carbaldehyde (HDTIC), HDTIC-1 and HDTIC-2, extracted from *Astragalus membranaceus* slowed down the telomere shortening rate of 2BS cells [44].

## 5. Conclusions

Using a cohort of healthy elderly donors with telomere shortening related to natural aging, we demonstrated that *A. mongholicus* Bunge HRE in vitro exposure could induce telomere elongation in circulating lymphocytes. This elongation was associated with less toxicity than danazol. This data could help define potential therapeutic strategies based on the use of this natural agent in populations with drastically short telomeres. It will be important to investigate the in vivo effects of the HRE in an elderly population with and without age-related diseases. The use of a specific model involving telomeropathy (such as TERT gene mutations, for example) could increase our knowledge relevant to the introduction of the tested HRE as a possible agent for counteracting telomere shortening and its consequences.

Association of this treatment with regular physical exercise, a healthy lifestyle, diet and lower exposure to stress could be a first step in slowing the natural aging process and combatting age-related diseases. A multidisciplinary approach and the definition of adequate experimental in vivo models are needed to win the battle against premature aging.

## 6. Patents

The patented process number is WO2001056584A1.

## Supplementary Materials

The following are available online at https://www.mdpi.com/2227-9059/8/2/31/s1. References [48,49,50,51,52,53,54] were cited in supplementary material.
