# Supplementary material for: The Use of Natural Agents to Counteract Telomere Shortening: Effects of a Multi-Component Extract of Astragalus mongholicus Bunge and Danazol
Source: Biomedicines. 2020 Feb 12;8(2):31. doi: 10.3390/biomedicines8020031 (PMC7168059; doi:10.3390/biomedicines8020031)
Supplement: Supplementary file 1 [file biomedicines-08-00031-s001.pdf]

Table S1. Chromatographic conditions for amino acids, astragalosides and flavonoids

	Detection of amino acids	Detection of astragalosides	Detection of flavonoids
Distance from lower edge of plate	8.0 mm	8.0 mm	8.0 mm
Distance from left and right edges of plate	20 mm	23 mm	25 mm
Space between bands	15 mm	13.5 mm	16.6 mm
Track number per plate	5	5	4
Development distance	50 mm	70 mm	70 mm
Development temperature	27.2 C	27.8°C	22.7°C
Development relative humidity	36.5%	35.5%	36.5%
Mobile phase	Butanol/ acetone/ acetic acid/ water (7/7/2/1) with 50.2 mg of ninhydrin	Ethyl acetate/methanol/water (50/6.75/5)	Ethyl acetate/toluene/formic acid (15/35/0.5)
Derivatization	Through heating to 100°C for 5 min	Through heating to 105°C for 3 min on a TLC plate heater and dipping (speed: 5, time: 0) in NP reagent then PEG reagent	No derivatization
Visualization	Under white light	Under UV light at 366 nm	Under UV light at 366 nm

NP: natural product; PEG: polyethylene glycol; TLC: thin layer chromatography; UV: ultraviolet

Table S2: Compounds identified using liquid chromatography with mass spectrometry (LC-MS) in the positive ion mode

No.	t_R	Compound	Formula	Mass	Ion m/z M+H theoretical	M+H (MS)	M+H (MS/MS)	Ref.	M+H standard or reference (MS/MS)
1	2.76	L-canavanine	$C_5H_{12}N_4O_3$	176.0909	177.0982	177.0979	(177)76/160/118/72/102	[49],[50]	76<br>76/118/190/102/72
2	2.99	asparagine	$C_4H_8N_2O_3$	132.0535	133.0608	133.0606	(133)74/87/116	[49],[51]	116<br>74/87
3	3.04	aspartic acid	$C_4H_7NO_4$	133.0375	134.0448	134.0446	88	[49]	88
4	3.26	glutamic acid	$C_5H_9NO_4$	147.0532	148.0604	148.0602	(148)84/102/130	[49,52]	102<br>84/102/130
5	6.57<br>ou 6.71	leucine	$C_6H_{13}NO_2$	131.0947	132.1019	132.1018	86	[49]	86
6	6.57<br>ou 6.71	isoleucine	$C_6H_{13}NO_2$	131.0947	132.1019	132.1018	86	[49]	86
7	11.19	calycosin-7-o-β-D-glucoside	$C_{22}H_{22}O_{10}$	446.1213	447.1286	447.1280	See negative ion mode		
8	13.47	ononine	$C_{22}H_{22}O_9$	430.1264	431.1337	431.1329	See negative ion mode		
9	14.95	calycosine	$C_{16}H_{12}O_5$	284.0685	285.0757	285.0751	See negative ion mode		
10	16.06	astragaloside IV	$C_{41}H_{68}O_{14}$	784.4609	785.4682	785.4668	See negative ion mode		
11	16.31	astragaloside III	$C_{41}H_{68}O_{14}$	784.4609	785.4682	785.4677	See negative ion mode		
12	17.27	astragaloside II	$C_{43}H_{70}O_{15}$	826.4715	827.4787	827.4775	See negative ion mode		
13	17.92	formononetin	$C_{16}H_{12}O_4$	268.0736	269.0808	269.0803	See negative ion mode		

Table S3: Compounds identified using liquid chromatography with mass spectrometry (LC-MS) in the negative ion mode
*M+HCOO-; STD: standard

No.	t_R	Compound	Formula	Mass	Ion m/z $[M-H]^-$ (theoretical)	Ion m/z $[M-H]^-$ (experimental)	Ion m/z $[M-H]^-$ (MS/MS)	Ref	Ion m/z $[M-H]^-$ standard or ref (MS/MS)
1	2.76	L-canavanine	$C_5H_{12}N_4O_3$	176.0909	175.0837	175.0830	See positive ion mode		
15	4.54	Saccharose	$C_{12}H_{22}O_{11}$	342.1162	341.1089	341.1088	(341)89/59/71/179/119/101/113	[53]	(341)71/89/101/179/59/113/119
16	6.36	citric acid	$C_6H_8O_7$	192.0270	191.0197	191.0189	(191)111/87/85	STD	(191)111/87/85
8	11.19	calycosin-7-O-β-D-glucoside	$C_{22}H_{22}O_{10}$	446.1213	445.1140	491.1199*	283/268	[54]	283
9	13.44	Ononine	$C_{22}H_{22}O_9$	430.1264	429.1191	475.1246*	267/252	[54]	267
10	14.95	Calycosine	$C_{16}H_{12}O_5$	284.0685	283.0612	283.0612	(283)268	[54]	268
11	16.06	astragaloside IV	$C_{41}H_{68}O_{14}$	784.4609	783.4536	829.4601*	(829)783/59/89/101/119/71/179/113/161	STD	(829)783/89/101/59/71/119/179/161/113
12	16.31	astragaloside III	$C_{41}H_{68}O_{14}$	784.4609	783.4536	829.4600*	873/71/101/113/85/161/59/489	[54]	161/101/489
13	17.27	astragaloside II	$C_{43}H_{70}O_{15}$	826.4715	825.4642	871.4705*	(871/825)59/89/101/179/71/161/766	[54	766/59/179
14	17.94	Formononetin	$C_{16}H_{12}O_4$	268.0736	267.0663	267.0664	(267)252	STD	252
17	18.51	astragaloside I	$C_{45}H_{72}O_{16}$	868.4820	867.4748	867.4750	59	[54	807/59

FigS1.

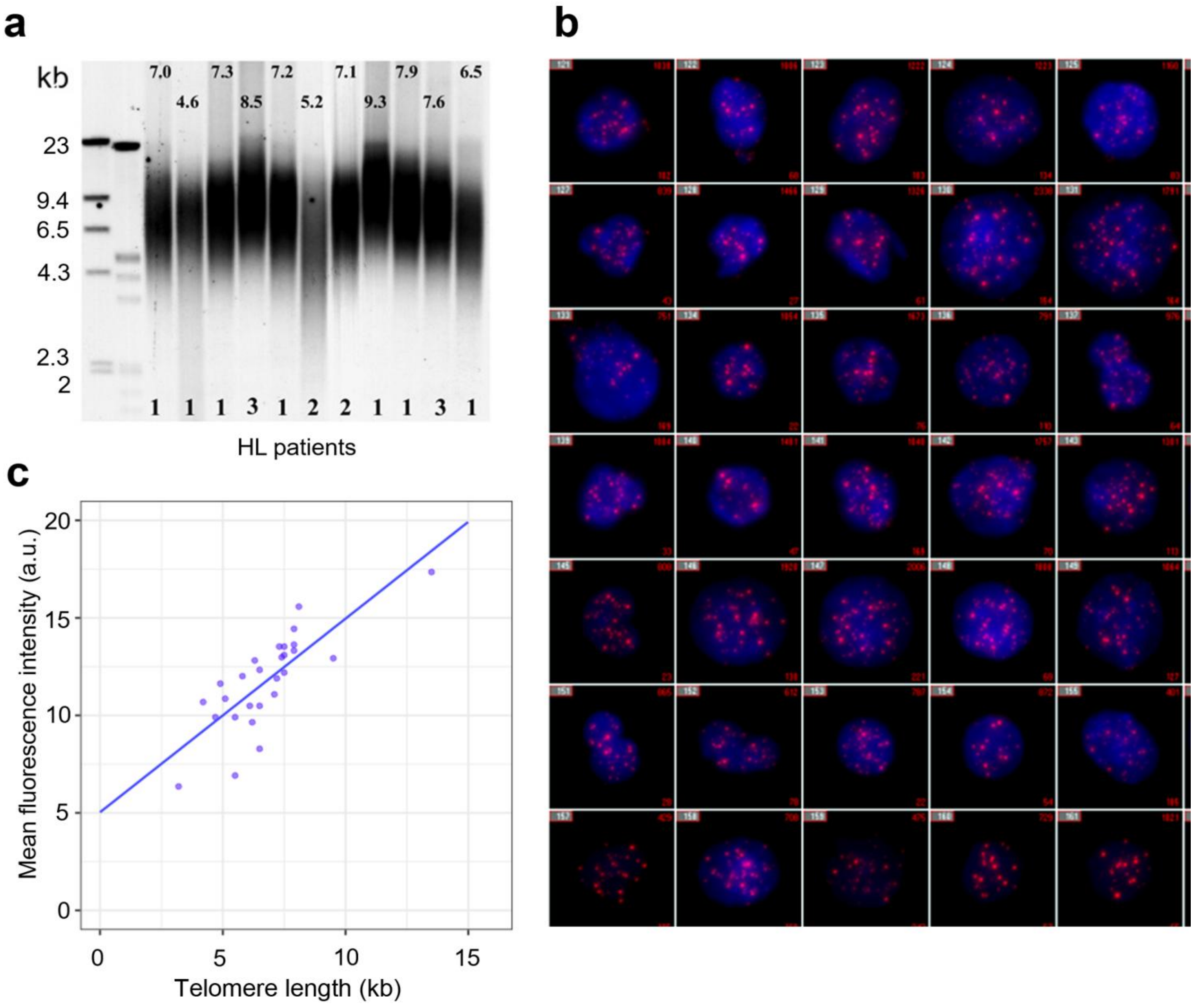
a
kb
23
9.4
6.5
4.3
2.3
2
7.0 4.6 7.3 8.5 7.2 5.2 7.1 9.3 7.9 7.6 6.5
1 1 1 3 1 2 2 1 1 3 1
HL patients
b
c
Mean fluorescence intensity (a.u.)
Telomere length (kb)
0
5
10
15
20

FigS2.

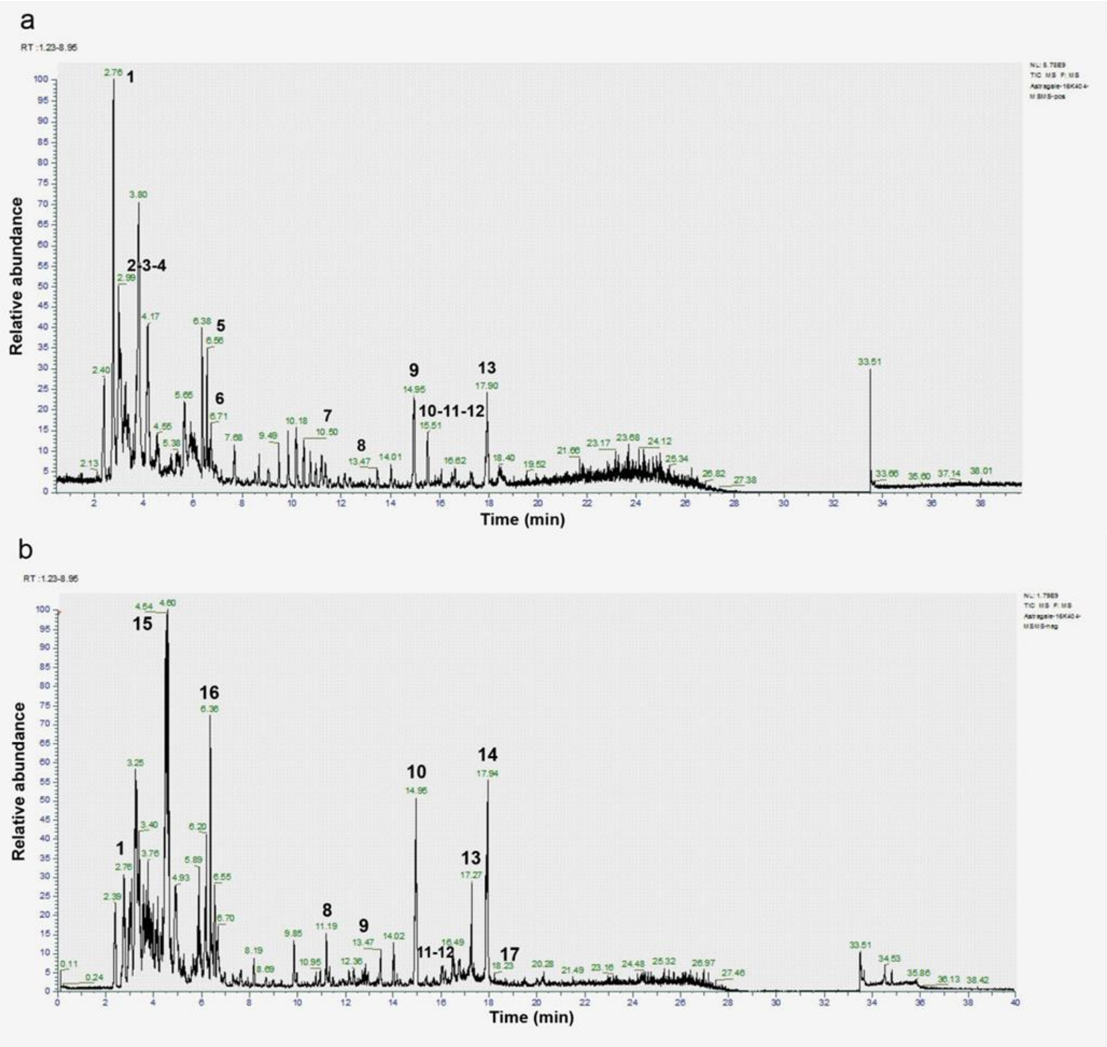

a
RT : 1.23-8.95
Relative abundance
Time (min)
1
2-3-4
5
6
7
8
9
10-11-12
13
b
RT : 1.23-8.95
Relative abundance
Time (min)
15
16
1
8
9
10
11-12
13
14
17

FigS3.

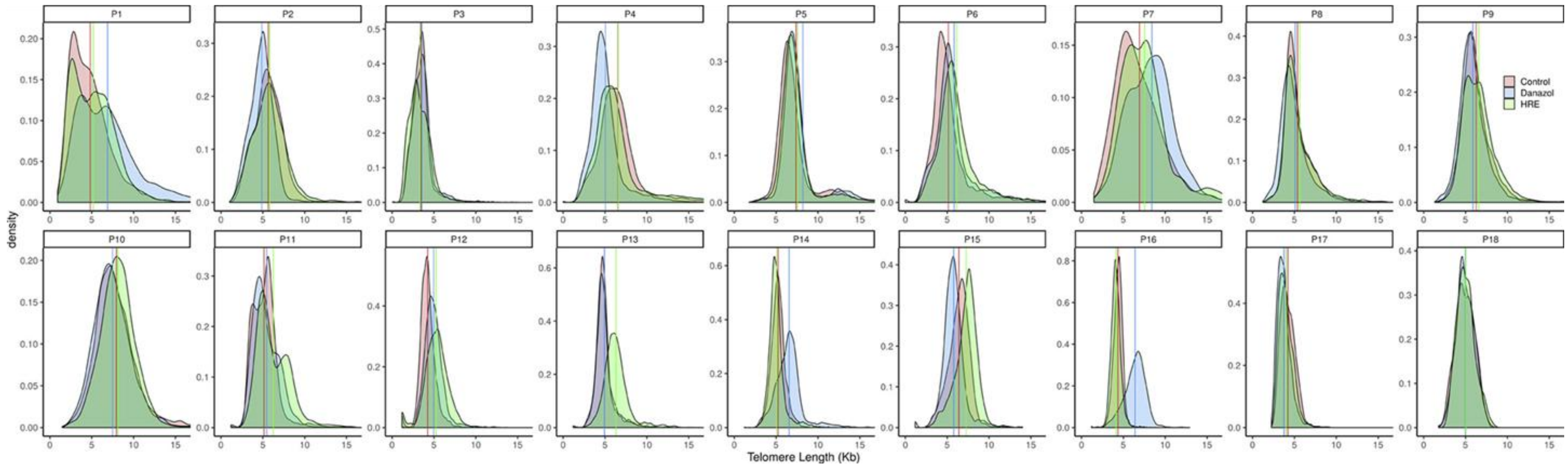
P1
P2
P3
P4
P5
P6
P7
P8
P9
P10
P11
P12
P13
P14
P15
P16
P17
P18
density
Telomere Length (Kb)
Control
Danazol
HRE
